# Responses of an Aphidophagous Ladybird Beetle, *Anegleis* Cardoni, to Varying Densities of *Aphis gossypii*

**DOI:** 10.1673/031.013.2401

**Published:** 2013-04-03

**Authors:** Omkar, Gyanendra Kumar

**Affiliations:** Ladybird Research Laboratory, Department of Zoology, University of Luc know, Lucknow-226007 (India)

**Keywords:** ECI, food exploitation, functional response, numerical response

## Abstract

Laboratory experiments were conducted to determine the functional and numerical responses of fourth instar larvae, adult male, and adult female ladybird beetles, *Anegleis cardoni* Weise (Coleoptera: Coccinellidae), to different densities of aphids, *Aphis gossypii* Glover (Hemiptera: Aphididae), on the bottle gourd, *Lagenaria vulgaris* Seringe (Cucurbitales: Cucurbitaceae). The results revealed a density dependent, Type II functional response of *A. cardoni*. Prey consumption increased curvilinearly with an increase in prey density for all three predatory stages. Numerical responses revealed significant increases in oviposition with increases in prey density. The food exploitation efficiency and the efficiency of conversion of ingested food decreased with increases in prey density. The attack rate was highest for adult females, followed by fourth instar larvae and adult males. Prey consumption was highest and handling time lowest in fourth instar larvae, followed by adult females and males. Therefore, fourth instar larvae of *A. cardoni* may be considered the most efficient predatory stage in aphid management strategies.

## Introduction

Coccinellids, commonly known as ladybird beetles, form an important group of biocontrol agents among insect predators due to their ability to feed on a variety of prey, e.g., aphids, mealy bugs, scale insects, whiteflies, thrips, and many more (Dixon 2000; Omkar and Pervez 2004). The effectiveness of predators depends upon their interactions with different prey species. To describe the nature of predation, Solomon (1949) proposed functional and numerical responses. Functional and numerical responses are used to assess the impact of a predator on the population dynamics of its prey (Murdoch and Briggs 1996). Functional response has been categorized into Type I, Type II, and Type III (Holling 1959), Type IV (i.e., dome shaped; Luck 1985; Bressendorff and Toft 2011) and Type V (i.e., negative exponential; Watt 1959; Sabelis 1992). Most studies reveal Holling's Type II functional response in coccinellids (e.g., Dixon 2000; Lee and Kang 2004; Pervez and Omkar 2005; Moura et al. 2006; Omkar and Pervez 2011), with only a few reporting Type III responses (e.g., Messina and Hanks 1998; Sarmento et al. 2007).

Disturbances while searching for prey and feeding can result in interference or stimulation. The number of prey killed increases with increases in prey density due to the interference-stimulation factor (Sandness and McMurtry 1970). All these factors may affect a predator's attack rate (a) and handling time (Th), which are the key factors of functional response. Evaluation of these two parameters is important when assessing the type of functional response. In addition to attack rate and handling time, the number of predators can also change as the number of prey increases, which is the numerical response of the preda-tor. This increase in predators may be because the predators aggregate in areas of high prey density, or because the reproduction and survival rate of predators increases. The aggregation of predators in response to changing prey resources may lead to higher attack rates by the predator. In nature, predators are known to respond both functionally and numerically towards their prey population, but functional response is known to be the key factor, as it is thought to be the determinant of numerical response (Keith et al. 1977). Therefore, the functional and numerical responses of a predator towards the changing density of a prey population are characteristics to evaluate the suitability of the predator.

**Figure 1.**
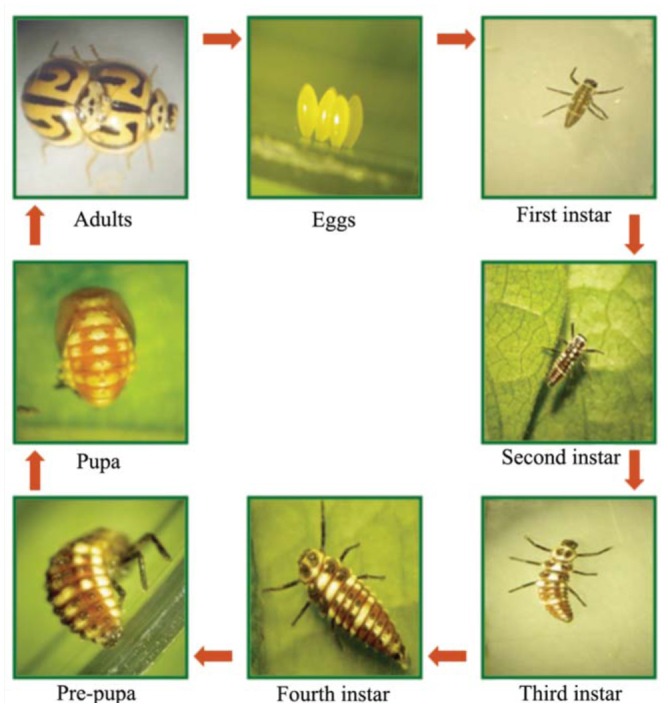
Life cycle of *Anegleis cardoni*. High quality figures are available online.

*Anegleis cardoni* (Weise) (Coleoptera: Coccinellidae) (Figure 1) is a medium-sized ladybird beetle, and is known to feed on a variety of aphids, e.g., *Brevicoryne brassicae* L. (Hemiptera: Aphididae), *Macrosiphum miscanthi* (Takahashi), *Macrosiphum pisi* Kaltenbach (Afroze 2000), as well as *Aphis* *gossypii* Glover, *Aphis craccivora* Koch, and *Lipaphis erysimi* Kaltenbach (Omkar et al. 2009) and many other aphids (Omkar et al. 2011). Futhermore, it has also been reported to prey on whiteflies (Ramani et al. 2002) and scale insects (Sundararaj 2008). Although previous studies have focused on the influence of prey quality and quantity on *A. cardoni* (Afroze 2000; Omkar et al. 2009; Omkar et al. 2010; Omkar et al. 2011), there is no information on prey-predator population dynamics, which is important in evaluating the performance of *A. cardoni* to a changing prey density and forecasting the suitability of the predator as a potential biocontrol agent. Therefore, this study was designed to investigate the functional and numerical responses of *A. cardoni* on its preferred aphid prey, *A. gossypii* under laboratory conditions.

## Materials and Methods

### Stock Maintenance

Adults of *A. cardoni* collected from trees of False Ashoka, *Polyalthia longifolia* (Magnoliales: Annonaceae), were grouped in Petri dishes to identify mating pairs. Thereafter, mating pairs were transferred to other Petri dishes (9.0 × 1.5 cm) and kept under controlled abiotic conditions (27 ± 2° C, 65 ± 5% RH, and 14:10 L:D photoperiod) in an environmental test chamber (ETC). Pairs were provided *ad libitum* with *A. gossypii* infested on the bottle gourd, *Lagenaria vulgaris* Seringe (Cucurbitales: Cucurbitaceae), together with host plant leaves collected from field. Egg laying and hatching were recorded twice a day. Precautions were taken so that larvae did not consume their egg chorions after hatching. Larvae were shifted into 250 mL, open-topped, cylindrical, glass beakers (9.5 × 6.5 cm) and kept in the ETC. Each beaker contained five larvae. Larvae were reared on*A. gossypii* until adult emergence. The aphid supply was refreshed every 24 hr. Newly emerged adults were reared in Petri dishes (same size as above) on the same aphid-host plant complex.

**Figure 2:**
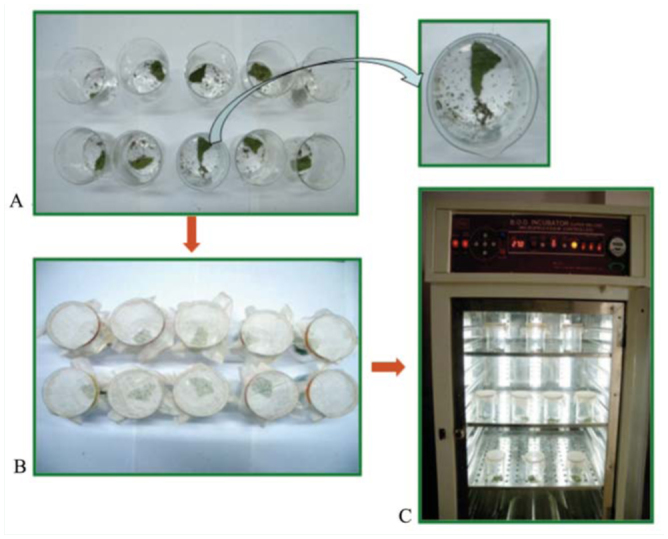
Experimental setup for functional response of fourth instar larvae of *Anegleis cardoni*. A: 320 third instar *Aphis gossypii*, one fourth instar *A. cardoni*, and a piece of *Lagenaria vulgaris* leaf per beaker (photo in zoom shows arena in a single beaker). B: beakers covered with muslin cloth and fastened with a rubber band. C: environmental test chamber. High quality figures are available online.

### Experimental Protocol

**Functional response.** Different predatory stages of *A. cardoni*, i.e., fourth instar larvae and 15-day-old, unmated, adult males and females were taken from stock culture and starved for 12 hr in the ETC under the previously mentioned abiotic conditions. Thereafter, they were kept separately in beakers (9.5 × 6.5 cm) and provided with variable numbers of third instar *A. gossypii* together with host plant leaves (Figure 2). Beakers were covered with muslin, fastened with a rubber band, and kept in the ETC. After 24 hr, predators were removed from beakers, and the number of unconsumed aphids was counted. The experiment was replicated ten times for each predaceous stage at different prey densities, i.e., 10, 20, 40, 80, 160, and 320. Aphid supply was not replaced during the experiment.

### Data Analysis

Functional response analysis involves determination of the type of functional response and estimation of the parameters of functional response curves (Juliano 2001). To distinguish between Type II and Type III responses, the shape of the functional response curve was analyzed with the help of a logistic regression (Juliano 2001) of the proportion of prey eaten (Ne) as a function of initial prey density (No). The data were fitted to a polynomial function using statistical software SAS (Version 9.0, http://www.sas.com/). The equation used was:
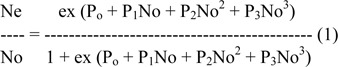

where, P_o_ (intercept), P_1_ (linear), P_2_ (quadratic), and P_3_ (cubic) are the parameters to be estimated. CATMOD procedure was used to estimate these parameters. The data were fitted to the equation below. Positive linear (P_1_ > O) and negative quadratic (P_2_ < 0) parameters indicate Type III functional response, while both parameters with negative values indicate Type II functional response. Thereafter, nonlinear least square regression procedure was used on SAS (Version 9.0) to estimate the Roger's (1972) random predator equation for Type II functional response:
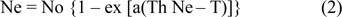

where, Ne is the number of prey consumed, No is initial prey density, a is the attack rate, Th is the handling time per prey, and T is the total time of exposure. This equation overcomes the problem of prey depletion. Statistically different parameter estimates were separated using asymptotic 95% confidence intervals (Juliano 2001). Type III functional response follows the equation:
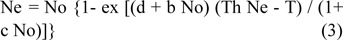

where b, c, and d are constants.

Data obtained from prey consumption by fourth instar larvae and adult females and males at different prey densities were subjected to one-way ANOVA, and means were compared using Tukey's honestly significant difference test at 5% levels using statistical software MINITAB- 2003 (http://www.min tab.com/) on a personal computer (PC).

### Numerical Response

In order to determine the numerical response, the method of Veeravel and Baskaran (1997) was followed. The methodology of aphid consumption by females in functional response analysis was further used for numerical response analysis. After aphid prey consumption, the ten females from each prey density were separated in Petri dishes and mated with a 15-day-old unmated male taken from stock culture. After mating, females were removed and placed in separate Petri dishes in the ETC with the same prey density as they had before mating. Oviposition by the females within the next 24 hr was recorded. Egg cannibalism by females was also taken into account by recording the presence of remnants of the eggs on the surface of Petri dishes to minimize the error. The experiment was replicated ten times.

The reproductive response of female *A. cardoni* at various prey densities was quantified (in terms of number) using the following indices:
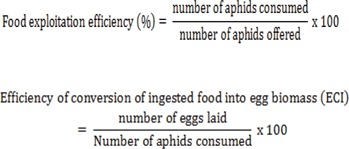


### Data analysis

Data obtained from the number of prey consumed, food exploitation efficiency, and food conversion efficiency at different predator densities were subjected to one-way ANOVA. Means were compared using Tukey's honestly significant difference test at 5% level using statistical software MINITAB- 2003 on personal computer (PC).

## Results

### Functional response

**Figure 3.**
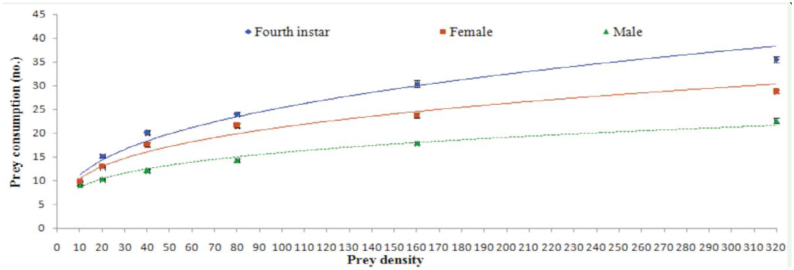
Prey consumption by different predaceous stages of *Anegleis cardoni* at different prey densities. Points with different colors are mean ± SE (n = 10 per stage). High quality figures are available online.

Prey consumption by the different predatory stages increased curvilinearly with increasing prey density (Figure 3). The number of prey consumed by the different predatory stages at different prey densities, i.e., 10 (F = 24.12, *p* < 0.0001, df = 2, 27), 20 (F = 78.77, *p* < 0.0001, df = 2, 27), 40 (F = 93.83, *p* < 0.0001, df = 2, 27), 80 (F = 148.20, *p* < 0.0001, df = 2, 27), 160(F = 115.59, *p* < 0.0001, df = 2, 27), and 320 (F = 174.24, *p* < 0.0001, df = 2, 27) was significantly different. Prey consumption on all prey densities (10, 20, 40, 80, 160, and320) was highest for fourth instar larvae (10.00 ± 0.00, 15.20 ± 0.33, 20.10 ± 0.46, 24.00 ± 0.42, 30.40 ± 0.75, and 35.50 ± 0.58, respectively) followed by adult females (9.80 ± 0.13, 12.90 ± 0.31, 17.60 ± 0.43, 21.60 ± 0.45, 23.70 ± 0.58, and 28.80 ± 0.36, respectively) and adult males (9.10 ± 0.10, 10.30 ±0.15, 12.10 ± 0.38, 14.30 ± 0.37, 17.90 ± 0.35, and 22.60 ± 0.50, respectively) (Figure 3). A comparison of means also revealed significant differences in prey consumption by different predatory stages at above prey densities.

**Figure 4.**
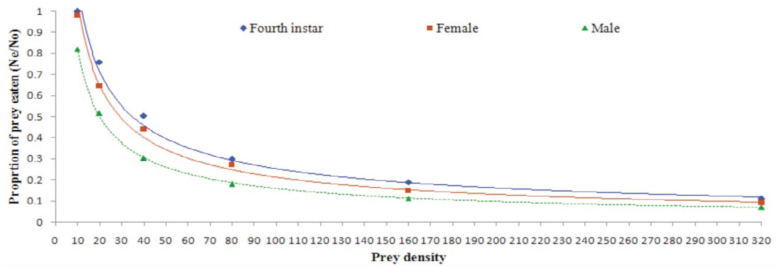
Proportion of prey eaten (Ne/No) by different stages (n = 10 per stage) of *Anegleis cardoni* at various prey densities. High quality figures are available online.

The results revealed a decline in the proportion of the number of prey consumed to the initial prey density (Ne/ No) with increasing prey density (Figure 4). Linear parameters (Pi) for all three predatory stages were found to be negative (P1 < 0), confirming a Type II functional response (Table 1). The coefficient of attack rate was found to be highest for adult females (0.00482), followed by fourth instar larvae (0.00418) and adult males (0.00385) (Table 2). Handling time was found to be longest for adult males (1.3478 hr) followed by adult female (0.9793 hr) and fourth instar larvae (0.7907 hr).

### Numerical response

**Figure 5.**
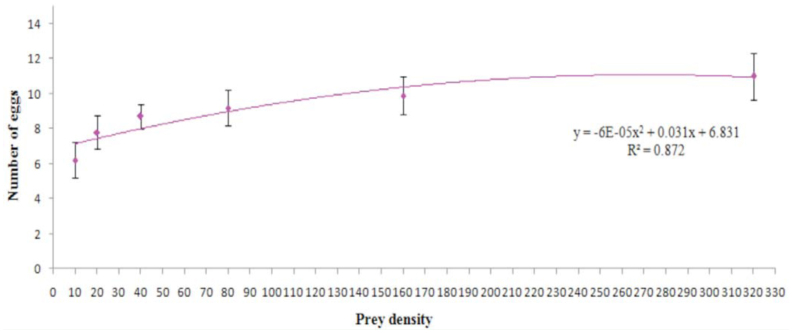
Number of eggs laid by females (n = 10) of *Anegleis cardoni* at various prey densities. Points are mean ± SE. High quality figures are available online.

Female *A. cardoni* laid the most eggs (11.00 ± 1.33) at the prey density of 320, followed by 160 (9.90 ± 1.10), 80 (9.20 ± 1.01), 40 (8.70 ±0.70), 20 (7.80 ± 0.94) and 10 (6.20 ± 1.01). Regression analysis revealed the number of eggs laid was positively correlated with prey density (Figure 5). The results revealed a curvilinear increase in number of eggs laid with increase in prey density (Figure 5).

**Figure 6.**
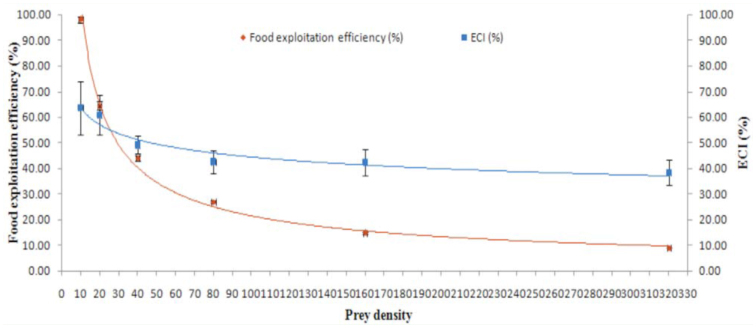
Food exploitation efficiency (%) and efficiency of conversion of ingested food (ECI) of females (n = 10) of *Anegleis cardoni* (in terms of number) at various prey densities. Points with different colors are mean ± SE. High quality figures are available online.

Prey density was found to significantly influence food exploitation efficiency (F = 1167.71, *p* < 0.0001, df = 5, 54) and the efficiency of conversion of ingested food (F = 2.64, *p* = 0.033, df = 5, 54). Both these indices decreased with an increase in prey density (Figure 6). Food exploitation efficiency and efficiency of conversion of ingested food were highest (98.00 ± 1.33 and 63.56 ± 10.50 respectively) at a density of ten aphids, and lowest (9.00 ± 0.11 and 38.38 ± 4.86 respectively) at density of 320 aphids. Individual means of these two indices also differed significantly from each other.

## Discussion

In this study, prey consumption by the three predatory stages of *A. cardoni* increased significantly with increasing prey density. However, the mean ratio of the number of prey killed (Ne/No) decreased curvilinearly. Attack rate was highest for adult females, followed by fourth instar larvae and adult male, whereas handling time was found to be longest for adult males, followed by adult females and fourth instar larvae. Linear coefficients, i.e. P1, for three predatory stages were also negative (Table 1). This result indicates that the predator showed a decreasing consumption rate with increasing prey density, thus confirming a Type II functional response. This finding coincides with Pervez and Omkar (2003), who found that aphidophagous coccinellid predators did not exhibit a high rate of prey consumption at higher densities.

Although several studies found that predators with a Type III functional response were efficient biocontrol agents (Holling 1965; Fernandez-Arhex and Corley 2003), most predators have been reported to exhibit a Type II functional response when successfully released on their prey (Timms et al. 2008; Ghosh and Chandra 2011). In a sigmoidal curve (Type III), a predator is assumed to learn how to circumvent some difficulties associated with prey capture (Schenk and Bacher 2002), whereas in a curvilinear shape (Type II), a predator is assumed to feed on prey without any initial delay in learning ways to circumvent difficulties (Sarmento et al. 2007).

The results revealed significant differences in the number of prey consumed among the three predatory stages of *A. cardoni*. Fourth instar larvae consumed the highest number of prey, followed by adult females and males. Thus, fourth instar larvae may be considered the most efficient predatory stage of *A. cardoni*. Higher predation by fourth instar larvae was probably due to their extra requirement of nutrients during their subsequent non-feeding pupal stage. The results also revealed that prey consumption by females was more than by males. This difference could be correlated with capture threshold, which depends upon the gut capacity of the predator; as males have a smaller gut capacity, they consume less food (Meiracker and Sabelis 1999).

Low handling time reveals the predator to be a good biocontrol agent. Handling time is proportional to the size of the prey; the larger the prey, the longer the time taken to consume it (Flinn et al. 1985). When females of *Cheilomenes sexmaculata, Propylea dissecta*, and *Coccinella transversalis* were provided separately with the aphid *Myzus persicae*, their handling times were 0.0043, 0.01, 0.0056 hr, respectively (Pervez and Omkar 2005). Mandour et al. (2006) reported that the handling time of fourth instar larvae and adult females and males of the ladybird beetle *Cydonia vicina nilotica* on the aphid *Aphis craccivora* was 0.0086, 0.0069, and 0.0115 hr, respectively. Omkar and Pervez (2011) found increased prey consumption when two heterospecific female ladybeetles were exposed together to different densities of *A. gossypii*.

The handling time was low (0.1582 hr) and the attack rate was high (0.00274) for the heterospecific combination in comparison to other conspecific combinations. However, in the current study, the handling time of *A. cardoni* was found to be much longer for all the predatory stages in comparison to these studies. The shortest handling time was found in the fourth instar larvae (0.7907 hr), followed by adult females (0.9793 hr) and males (1.3478 hr), whereas attack rate was highest for adult females (0.00482), followed by fourth instar larvae (0.00418) and adult males (0.00385). The longer handling time may be size-based. *A. cardoni* is a smaller-sized lady beetle, and *A. gossypii* may be too large aphid for quick consumption. Furthermore, its developmental duration is longer and its reproductive rate is much lower (Omkar et al. 2009) than the above-mentioned ladybeetles. A low reproductive rate of a predator reflects a low food requirement, which may be correlated with poor food utilization efficiency. In the case of all three predatory stages in the present study, prey consumption increased with increases in prey density. This result is probably due to the differences in the area of discovery. At lower prey densities, aphids were widely distributed, which increases the time it takes for a predator to come across a prey, whereas at higher prey densities the amount of time it takes to find a prey is shorter (Hodek 1967).

Prey consumption and oviposition by females increased with increases in prey density, whereas food exploitation efficiency and efficiency of conversion of ingested food decreased with increases in prey density. This difference is possibly due to the fact that an increase in food quantity facilitates the development of more numbers of ovarioles (Evans 2000). Honek (1978) reported that the variation in ovarioles maturation was positively correlated with aphid densities, hence affecting the egg production by the predator. At a certain prey density, when a predator has consumed sufficient prey, it gets satiated and thereby tends to decrease feeding. Previously starved females initially allocate and consume aphids to make up the weight loss during the starvation; however, after being satiated, they consume a certain number of aphids only to oviposit and to cover the cost of metabolism (Isikber 2005).

In conclusion, the data from the present study provide information as to how *A. cardoni* responds to changes in prey density under laboratory conditions. Fourth instar larvae had the highest prey consumption and the lowest handling time; therefore, fourth instar *A. cardoni* may be considered as the superior predator of *A. gossypii* in comparison to adults. Oviposition by females increased with increases in prey density, while efficiency of conversion of ingested food decreased. This study revealed that prey density has a significant influence on prey consumption, and if the prey searching rate is increased, the prey consumption will decrease. Furthermore, *A. cardoni* had a longer handling time and lower prey consumption in comparison to many ladybird beetles. However, these results are based on a laboratory study. As prey consumption depends upon the habitat complexity and prey-predator densities, evaluation of the potential of *A. cardoni* together with heterospecifics in the laboratory and also in the field in its natural habitat is needed to further understand the foraging behavior and preypredator interactions.

**Table 1.**
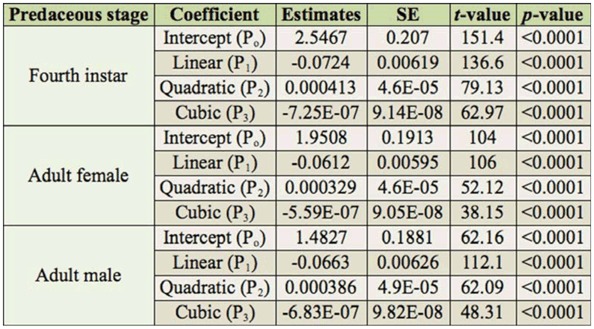
Procedure CATMOD analysis of maximum likelihood estimates from logistic regression of the proportion of prey killed by different stages of *Anegleis cardoni* as a function of initial prey density.

**Table 2.**
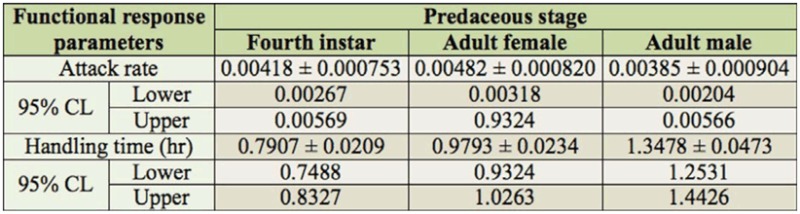
Functional response parameter estimate values (± SE) of attack rate (a) and handling time (Th) at 95% confidence limit (CL) for different stages of *Anegleis cardoni* obtained by least square method (PROC NLIN).

## Abbreviations:

ETC,: environmental test chamber
